# Supplementary material for: Are carbon emissions trading and green financial instruments synergistic? -Comprehensive quantitative research based on content analysis
Source: PLoS One. 2024 Mar 7;19(3):e0298601. doi: 10.1371/journal.pone.0298601 (PMC10919601; doi:10.1371/journal.pone.0298601)
Supplement: S1 Data — (ZIP) [file pone.0298601.s001.zip › Data/地级市Z_F工作报告.zip!/║┌┴·╜¡/╞δ╞δ╣■╢√╩╨/└┤╫╘╣1⁄2╓┌║┼í╛┬φ┐╦╩2╛▌═°í┐_2011.docx]

各位代表：

现在，我代表市人民政府，向大会作工作报告，请予审议，并请市政协各位委员提出意见。

一、2010年工作回顾

2010年，我们在市委的领导下，圆满完成了市十四届人大四次会议确定的各项任务，胜利实现了“十一五”规划目标。“十一五”期间，是改革开放以来我市发展最好最快的五年，综合经济实力显著增强，各项社会事业长足发展，城乡环境面貌明显改观，人民生活水平日益提高，为全面建设小康社会打下了坚实基础。

2010年，预计完成地区生产总值830亿元，同比增长17%，“十一五”期间年均增长13%。财政总收入突破百亿元，达到110.5亿元，增长42.5%，是2005年的3.2倍，年均增长26.4%；地方财政收入78.7亿元，增长54.9%，是2005年的3.7倍，年均增长30.1%。全社会固定资产投资完成476亿元，增长40%，是2005年的4.8倍，年均增长37%。

——工业经济企稳提速。按照壮大传统产业和培育新兴产业的要求，全力抓好项目建设，促进了工业经济快速发展。实施重点推进项目120个，超亿元项目95个，完成投资149亿元。其中工业项目89个，超亿元项目65个，完成投资95.6亿元。2010年是我市近年来项目建设数量最多、质量最好、速度最快、力度最大的一年。老企业升级改造步伐加快，综合实力和发展后劲明显增强，昊华“双三十”、两大机床主导产品产业化、博天糖业技术改造、安泰生物产业化、泰来油田新打油井等项目建成投产，一重大型铸锻件生产流程改造、北钢年产70万吨钢水、齐轨道铁路货车产品升级、华鹤节能阻燃门产业化、飞鹤乳制品扩能、博城糖业扩建等项目加快建设。装备工业配套能力有了新提高，腾翔30万吨铸锻造加工基地启动建设，宇通高档数控转台和铣头产业化项目加快推进，新增机械装备配套企业10户。坚持定向定位招商，落实5000万元以上工业项目84项，到位资金101亿元。成功引进了黑龙江龙华新能源汽车、中国一拖大农机、中粮60万吨玉米深加工三个超20亿元的大项目，对于全市工业结构调整和发展加速具有重大的战略意义。克山曙光、富裕二期风电项目建设完成，大唐碾子山风电场设备采购完毕。启动实施了园区建设“15211”工程，齐齐哈尔高新区晋升为国家高新技术产业开发区，富拉尔基工业园区新入驻企业15户，北苑工业园区新上了2个超15亿元的大项目，铁锋、昂昂溪、富裕、依安等工业园区发展加快，承载和吸引项目的能力明显增强。科技创新取得新成果，获国家科技进步二等奖1项、省科技进步一等奖4项。高新技术企业实现产值340亿元，增长20%。我市被命名为“国家商标战略实施示范城”、“国家新型工业化装备制造产业示范基地”和“中国木门制造基地”。规模以上工业完成增加值290亿元、主营业务收入882.4亿元、利税总额107.2亿元，分别增长41%、46.8%和40％。工业经济效益综合指数达到270，提高60点。

——农业发展成绩喜人。以农民增收为目标，大力发展现代农业，不断提高农业经营组织化程度，加快了农业科技化、规模化和产业化进程。实现农业总产值352亿元，增长8%。粮豆薯总产193.6亿斤，增长16.8%，创历史新高。土地规模经营达到1149万亩，增长31.2%，占村集体耕地面积的38.2%。绿色和有机作物种植面积达到1200万亩，增长33%。兴十四现代农业示范园区一期工程达到省级标准，县级农业科技园区达到44个，对农业增产增收发挥了重要的示范和引领作用。畜牧业在市场低迷形势下稳步发展，主要畜禽品种饲养量持续增长。出台了加快发展奶牛业的实施意见，奶牛存栏55.5万头、增长11%，居全省第一，其中规模养殖16.5万头、增长30%，单产比非规模养殖提高25%；鲜奶总产量132万吨，增长7%。农业产业化步伐进一步加快，投资89.8亿元新建、续建了农产品精深加工项目28项，市级以上龙头企业达到190户，销售收入增长31%。农机等专业合作社蓬勃发展，争取千万元现代农机专业合作社101个，占全省的31%，田间综合机械化程度达到87.5%。农业基础设施建设得到加强，投资3.5亿元实施了14个重点水利项目，三大灌区节水改造、中北引扩建、泰来抗旱灌溉引水工程和水库除险加固工程等进展顺利。新增水田35.4万亩、抗旱灌溉面积57.6万亩。投资4175万元，治理水土流失45.27万亩。实施了总投资12亿元的土地整理工程，完成投资近5亿元，新增耕地6.2万亩。累计转移农村劳动力103万人，实现劳务收入71.5亿元。农村居民人均纯收入突破6000元，增长25%以上。

——第三产业繁荣活跃。着力提高第三产业发展质量和服务能力，完成增加值316.2亿元，增长11.6%。以提高扎龙景区旅游吸引力和带动力为重点，高水准规划推进了扎龙景区、温泉小镇、齐扎公路升级改造及沿线旅游节点、昂昂溪俄罗斯风情小镇等项目，罗西亚大街被评为“中国十大历史文化名街”。牵头成立了“大庆、齐齐哈尔、黑河城际旅游联盟”，推出了三市旅游一卡通，提升了“情系黑土地”旅游线路的吸引力。谋划了综合物流产业园区和各类专业市场等项目，铁锋汽贸城项目开工建设。成功举办了第十届绿博会、第七届小交会和第九届关东文化旅游节。餐饮、娱乐等传统服务业日益兴旺，汽车、家电、电子等商品持续热销。社会消费品零售总额实现365亿元，增长19%。对外贸易逆势攀升，进出口总额完成8.82亿美元，增长30%。金融业发展速度和质量双提升，支持地方经济发展能力明显增强。发放各项贷款541.2亿元，增长21.5%，其中大项目建设及重点企业贷款98亿元，增长20.4%；中小企业贷款154亿元，增长26.4%；涉农贷款222亿元，增长22%。龙江银行齐分行县域支行网点全面铺开，小额贷款公司发展势头良好。城投公司的投融资主体地位得到强化，新建了中小企业和畜牧业融资平台并发挥积极作用。一重集团成功上市。

——城乡建设全面升级。按照市委城乡建设工作总体要求，转变城市规划理念，出台了城市规划控制八项规定，明确了城市道路、楼宇、绿化、亮化、停车设施、小区配套等建设标准，为建设高品位的城市提供了刚性保障。对38项规划进行了审查调整，回购已出让的5.4公顷建设用地用于建设7处街头绿地公园；对14栋临湖、临中心商业区建设密度较大、容积率较高的高层建筑进行了降层处理；对个别违规超高建筑进行了强制拆除。高水准编制了滨水城市建设总体规划、齐齐哈尔高新区总体规划及劳动湖南扩、哈齐客运专线南站区域、中华路总体风貌改造等专项规划。居住条件进一步改善，市区新建商品房160万平方米，拆迁城市棚户区139万平方米、建设回迁房146万平方米，改造低温楼4.7万平方米，改造农村泥草房3.98万户、235.8万平方米。公路建设实现历史性突破，4条高速公路建成通车，新建跨江大桥3座，改造城区道路29条，新修农村公路3405公里。“三供两治”项目开工建设28项，完成投资9.68亿元。经过180天艰苦奋战，中心城区污水处理二期工程和富拉尔基污水处理工程实现通水试运行，日处理污水能力达到30万吨。列入松花江流域水污染防治规划的项目竣工8项，11个项目全部完成，每年可削减化学需氧量3万多吨。改造污水管网1.3万延长米，实现了中心城区污水和雨水排放分离。日处理1200吨的垃圾处理场项目取得积极进展。建设了天然气复线工程，供气能力提高了23倍。大力推进10个省级试点镇和 14个市级试点镇规划建设，撤并自然屯33个，村镇面貌有了新变化。城市管理成效显著，明确了市、区两级城市管理职责，初步建立起“7+1”城市管理相对集中行政处罚权体制，加强了城管执法力量，形成了城市管理新格局。下大力气对浏园景区私搭滥建、火车站广场秩序和周边建筑、圣弥厄尔教堂周边和出城口环境进行了专项整治，集中开展了牌匾广告整顿，为城市环境综合治理起到了示范作用。城区绿化取得新突破，突出常绿树种和“大树进城”，高起点搞好联通大道、冬韵园等9个重点地块的绿化设计；投资1.6亿元，植树201万株，新增城区绿地1306公顷，城区绿化覆盖率达到40%。植树造林2.83万公顷，增长15%，造林绿化三年会战任务如期完成。新晋升3个省级自然保护区。全面完成了节能减排目标，万元GDP综合能耗同比下降5%,削减化学需氧量12193吨、二氧化硫1984吨。第二轮土地利用总体规划大纲获国家批准，争取到批次用地指标242公顷，是前5年的总和，保障了项目建设用地需求。切实加强了建筑用砂石开采整顿和税费征管工作，收缴税费4430万元，是上年的3倍。

——民生工程扎实推进。以保障改善民生为重点的16件实事基本完成，居民生活水平进一步提高。城镇居民人均可支配收入达到1.3万元，增长8.5%。实施更加积极的就业政策，开发公益岗位4622个，发放岗位补贴1694.7万元；实现城镇新增就业12.7万人，下岗失业人员再就业11.4万人，城镇登记失业率控制在3.9%以内。社会保障水平有了新提高，新增“五险”参保人员38万人。被确定为全省“城镇化进程中养老保障制度体系构建试点城市”，出台了“城中村”被征地农民转为城镇居民后社会保障办法。全省新农保现场会在我市召开，依安县荣获全省首批新农保试点工作先进单位。深入实施医药卫生改革，10个县区实行了基本药物零差价销售，居民用药价格降低37%。出台了参保人员转诊转院试行办法，保证了患者就诊享有优先选择权。市区社区卫生服务中心全部纳入了医保支付范围，两次调低了低保人员住院起付线，取消了灵活就业人员参加医保疾病限制，启动了城镇居民基本医疗保险门诊统筹，提高了城镇居民特定疾病补助医疗保险年最高支付限额，使45万人受益。开展了新农合门诊统筹，增加了门诊大病、慢病补偿病种，提高了补偿比例及封顶线，农村常住人口参合率达到99.8%，高于全省平均水平2个百分点。城乡低保应保尽保。9350户棚改居民迁入新居，建设廉租住房27.1万平方米、5885套，为22848户“双困”家庭发放补贴4935万元。落实帮扶资金2亿元，农村贫困人口下降8%。为1000名贫困白内障患者实施了免费复明手术，为500名贫困精神病患者提供了免费医疗救助，帮助农村530户贫困残疾人家庭新建和改建了住房。教育工作取得新成绩，新增标准化学校71所，改造危房校舍71.8万平方米，“校安工程”被省确定为示范工程，办学条件进一步改善。义务教育均衡发展，有4个县区进入全省“双高普九”行列。素质教育深入实施，教育质量稳步提高，普通高考录取率达到87.1%。中职招生连续四年突破2万人，毕业生就业率保持在95%以上，人均劳动报酬提高了10%。高等教育加快发展，齐齐哈尔职业学院专升本工作通过省级评审。完善了公共文化服务功能，文化惠民活动广泛开展，城乡居民文化生活日益丰富。深入开展了全民健身活动，积极参加重大体育赛事并取得较好成绩。继续保持低生育水平，人口素质进一步提高。

——社会管理得到加强。强化治安基础建设，规划实施了城市监控系统全覆盖、智能卡口监控系统等项目，治安防控信息化水平明显提升。加大了社会治安综合治理力度，深入开展了严打整治专项行动，刑事案件稳中有降，群众安全感进一步增强。严格落实企业主体责任和政府监管责任，扎实推进企业安全标准化升级达标，切实加强道路交通事故预防和管理，努力构筑社会消防安全“防火墙”工程，各类安全事故下降了26.8%。畅通群众诉求渠道，化解信访难案积案，信访总量进一步下降。强化了药品、食品、农资等市场监管，规范了市场价格和收费秩序，严厉查处了不正当竞争、商业贿赂、违法广告、制假售假等扰乱市场经济秩序行为。社区建设成效显著，制定了社区公益用房三年发展规划，对新社区按建筑面积的2‰配建，对未达标的老社区明确了达标时限，新建和改扩建社区公益用房1.35万平方米。第三届城市社区换届圆满完成，工作人员平均年龄39.5岁，待遇有了新提高。开展了百项服务进社区活动，全面推进居家养老服务，为2.1万名高龄老人发放生活补贴1272万元，我市成为国内首个“国际老年友好城市”。建立了物业企业信用考评体系，中心城区小区业主委员会组建率达到70%。

——行政效能明显提升。坚持依法规范廉洁行政，对行政权力进行了确权，围绕行政审批、工程建设、土地出让、财政资金使用、服务支持项目建设等整章建制，制定并实施了政府投资项目招标投标监督暂行办法、建设工程行政审批行政过错处理暂行办法、重点项目推进工作考核办法、企业评议政府部门和“站所队”工作实施方案等制度规定，规范了行政权力运行，优化了审批流程，大幅缩短了审批时限。拓展了审计领域，开展了领导干部“生态审计”、建设用地容积率、政府投资工程预算和企业税费缴纳等专项审计。加大了政务公开力度，实施了网上公开和咨询。开设了电视直播互动栏目《关注?沟通》，为群众解决热点难点问题467件。机构改革积极稳妥推进，理顺了政务服务中心、市区城管等管理体制。加强了政府法制建设，起草、审修地方性法规（草案）7部、政府规章9部，清理规范性文件3563件。“五五普法”圆满完成，依法治市取得良好成效。

我市第六次全国人口普查工作顺利进入录入阶段。荣获全省双拥模范城“七连冠”。关心下一代工作进入全国先进行列。外事侨务工作和接待水平进一步提高。公共机构节能工作成效明显。民族宗教、档案、地方志、人防、气象、民兵预备役等工作取得了新成绩。

各位代表，过去的一年，我们把加快发展作为政府工作的主线，坚决贯彻市委的总体部署，不错失历史的机遇，不耽搁发展的脚步，牢牢把握发展关键，充分发挥比较优势，千方百计上项目，全力以赴保增长，老企业实力明显增强，新产业实现重大突破，全市经济进入了结构深度调整、速度质量显著提升的发展时期。

过去的一年，我们把谋划长远作为政府工作的基点，在抓好当前发展和保证群众现实需求的同时，面向未来，打牢基础，用长远眼光谋划产业发展和城乡建设，用战略思维规划园区布局和城市新区，用可持续的政策措施保障和改善民生，为今后发展积攒了后劲。

过去的一年，我们把改革创新作为政府工作的动力，渗透到各个环节，改革工作方法，创新工作机制，规范工作流程，堵塞工作漏洞，在创新中解决历史难题，在规范中追求公平正义，进一步完善了民主科学的决策机制、高效有力的推进机制、维护公平的保障机制，提高了政府工作的规范化、制度化水平。

过去的一年，我们把破解难题作为政府工作的突破口，敢于直面问题，勇于攻克难关。通过帮助企业解决用地、融资等难题，为企业加快发展铺平了道路；通过加大整治城市重点部位力度，为实现城市规划建设目标扫清了障碍；通过采取超常规措施服务大项目建设，营造了优良的发展环境。

过去的一年，我们把抓好落实作为政府工作的基本功，以咬定青山不放松的精神，对看准的事、决定的事，精心组织，狠抓推进，跟踪问效，务求实效，进一步转变了政府公务人员的工作作风，提高了政府部门的工作效能，形成了凝心聚力、干事创业的良好氛围。

各位代表！全市经济社会发展取得的可喜成绩，得益于市委的坚强领导，得益于市人大、市政协及各民主党派、社会各界的监督支持，得益于驻齐中、省直单位的鼎力相助，得益于全市人民的团结奋斗。在此，我代表市政府，向全市人民，向所有为我市经济社会发展做出贡献的同志们、朋友们，表示崇高的敬意和衷心的感谢！

我们也清醒地看到，全市经济社会发展中还存在一些困难和问题。新兴产业刚刚起步，附加值高的终端市场产品少，牵动经济发展的大项目不多，民间资本参与产业发展不够踊跃。农业产业化龙头企业整体规模偏小，品牌农产品的商品量不大。旅游景点建设水平有待提升，物流、文化等服务业发展滞后。城市基础设施还需完善，农村城镇化水平不高，社区管理和服务有待进一步加强。有些政府公务人员思想不够解放，市场意识、效率意识、群众意识淡薄；有的政府部门创新能力不强，管理社会、促进发展、服务群众的有效方法不多。对此，我们一定高度重视，认真加以解决。

二、2011年工作任务

2011年是“十二五”开局之年，也是加快全市经济社会发展的关键之年。市委十一届十次全会绘就了未来五年全市经济社会发展的宏伟蓝图。到2015年，实现地区生产总值1660亿元，年均增长15%；财政总收入突破200亿元，年均增长15%；全社会固定资产投资1800亿元,年均增长30%以上；城镇居民人均可支配收入2.3万元,年均增长12%；农民人均纯收入1.1万元，年均增长13%。面对又一个加快发展的“黄金五年”，我们要以奋发有为的精神状态和求真务实的工作作风，全力打造“滨水园林城市、绿色食品之都、装备工业基地、生态旅游胜地、历史文化名城”，努力把齐齐哈尔建设成为产业实力更加雄厚、城市特色更加突出、生态环境更加优良、人民生活更加富足、社会更加安定和谐的创业宜居城市。

今年工作的总体思路是：坚持以科学发展观为统领，以经济总量翻番为目标，以转变发展方式为主线，以大项目建设为支撑，深入落实市委十一届十次全会精神，强化一个基础（农业），打好两个攻坚战（工业振兴、三产升级），培育六大产业（装备、食品、化工、新能源、新材料、新生物医药），推动社会事业全面进步、城乡面貌明显改观、人民生活持续改善，努力实现经济社会更好更快发展。主要预期目标是：地区生产总值增长13%，地方财政收入增长16%，全社会固定资产投资增长30%，规模以上工业增加值增长15%，社会消费品零售总额增长16%，城镇居民人均可支配收入增长10%，农村居民人均纯收入增长13%，居民消费价格总水平控制在4%左右，城镇登记失业率控制在4.3%以内，万元GDP综合能耗下降5%以上。

（一）以齐齐哈尔国家高新区和富拉尔基工业园区为龙头，以大项目建设为支撑加快实施“15211”工程，推进工业经济总量扩张和发展方式转变。举全市之力建设好国家高新区。齐齐哈尔高新区晋升为国家高新区，为我市推进经济转型、实现翻番战略注入了巨大动力。按照国家要求，抓紧理顺高新区管理体制和运行机制，修订完善高新区建设发展规划和政策体系。10月末前全面完成征地及主要道路和水、电、气、热等基础建设，启动建设专业孵化器、中试基地、金融街、高端人才居住园区。重点引进和培育高端装备制造和食品加工等高新技术企业，加快推进以新能源汽车产业园为重点的“三新”产业发展，鼓励大学科技园发展和产学研结合，吸引重点实验室、工程技术中心、企业总部和研发中心、信息技术企业等进入园区，使高新区逐步成为引领产业发展、提高自主创新能力、促进经济结构调整与发展方式转变的强大引擎和重要的经济增长极。今年高新区固定资产投资力争达到30亿元，到2015年销售收入达到500亿元。提升富拉尔基工业园区综合竞争力。依托富拉尔基区雄厚的工业基础和人才技术优势，以国际化视野和市场化手段，把富拉尔基区打造成为全省装备制造业集中建设区和国家高端装备制造业研发生产基地。重点发展以北钢、黑化为依托的500亿元钢铁化工产业集群，以一重为依托的300亿元装备制造产业集群，以蒙西集团为投资主体的100亿元新型建材产业集群，以石油天然气开发、风力发电、生物质发电等为依托的100亿元能源开发产业集群??，到2015年销售收入达到1000亿元。今年固定资产投资力争达到62亿元，新上亿元以上项目10个。搞好富拉尔基工业园区与高新区的协作分工，推进两个园区相互支撑、互动发展，形成拉动全市经济发展的两大龙头。加快北苑工业园区建设，以东方红工业园、蒙牛、腾翔三大项目为依托，吸引更多的相关项目和配套企业入驻，延长产业链条，促进现代农机制造和绿色食品产业集群化发展。同步加快昂昂溪、铁锋、富裕、依安、龙江、讷河等重点园区及其他园区建设，整体推进“15211”工程加快实施。全力推进重点产业项目建设。把2011年确定为项目建设年，重点推进200个以上投资超5000万元项目建设，其中产业项目173项，年度计划投资128亿元。促进已投产项目尽早达产达效，抓好昊华“双三十”、齐二机床高档数控机床产业化等项目后续服务工作。推进已开工项目尽快投产，中粮60万吨玉米深加工项目力争6月投产，新能源汽车产业园项目确保7月整车下线，一拖东方红工业园项目确保8月产出第一台大马力拖拉机。加快推进一重大型铸锻件生产流程改造、齐轨道铁路货车产品升级、北钢500万吨钢和450万吨材、齐重数控重大装备产业化基地、腾翔30万吨铸锻造加工基地、宇通高档数控转台和铣头产业化、飞鹤乳制品扩能、风力发电等项目建设。大力支持装备制造、食品加工、化工等传统产业升级改造，积极引进“三新”产业项目。注重开发终端市场产品，优化工业产品结构。依托蒙东地区丰富的煤炭资源和大庆及本市的油气资源，加快煤炭和油气资源深加工产业发展。争取恢复启动昊华炼油生产，并延伸生产精细化工产品。围绕骨干企业和大项目，积极引进和谋划配套产业项目，确保配套产业增加值增长30%以上。加快发展循环经济，大力推进重点企业节能减排项目建设，积极争取国家“十城千辆”节能与新能源汽车示范试点城市。全方位服务企业发展。加强与国家和省有关部门及企业总部的联系，加大企业项目和资金争取力度。加强政企沟通，完善项目建设考评机制。坚持大项目建设领办代办制度，对投资超亿元项目实行市级领导全程包保，确保无障碍建设。科学搞好土地储备，保证项目建设用地供应。搭建高水平的融资服务、信息支持、产品展示和协作交流平台，为企业生产经营提供服务。支持企业实施品牌战略和重组扩张，增强发展实力。完善支持民营企业发展的政策措施，专门设立中小企业办事窗口，实行“一站式”服务，扶持一批社会声誉好、发展潜力大的中小企业做大做强。

（二）大力发展现代农业，促进农产品生产大市向加工大市转变，加快城乡一体化进程。提高粮食产能和市场竞争力。加快松嫩平原农业综合开发试验区齐齐哈尔项目区和粮食产能工程建设，走内涵挖潜、提高单产之路，确保粮食产能稳定在170亿斤以上。抓好国家级绿色食品原料标准化基地建设，新建绿色食品原料基地100万亩。发挥杂粮生产的比较优势，建设全国杂粮产业基地，提高杂粮品质，扩大生产规模，签订销售订单，搞好深加工和精包装，打进大城市、大市场、大超市，杂粮种植面积达到120万亩，增长39.5%。大力发展牧草和经济作物，牧草面积达到4万亩，增长10%，经济作物面积达到195万亩，增长8.3%。加快兴十四现代农业示范园区建设，尽快建成国家级现代农业示范园区，发挥更大的示范引领作用。实施畜牧业升级工程。通过政府引导和政策扶持，提高畜牧业组织化程度，全面推进奶牛、肉牛、生猪等畜禽品种的标准化养殖和产业化经营。继续建好大型现代化牧场，重点推广奶牛专业合作社、家庭牧场、规模养殖场和养殖小区等模式，培育发展更多的规模养殖主体和典型示范群体，促进奶牛存栏总量和质量进一步提升。力争奶牛存栏达到62万头、增长11%，其中规模养殖20万头、增长21.2%；鲜奶产量达到145万吨，增长9%。加快农业产业化进程。因地制宜推进土地规模经营，力争达到1500万亩，建设好农业产业化基地。发挥“中国绿色食品之都”的品牌优势，积极引进大型龙头企业对农畜产品进行精细化、系列化开发，延伸壮大乳品、肉类、玉米、水稻、马铃薯、甜菜、大豆、杂粮和蔬菜等重点产业链。加大农产品品牌整合力度，着力打造知名品牌，扩大品牌农产品商品量，达到农产品总产量的20%以上。积极培育专业购销大户、经纪人、代理商、批发商等农产品经销主体，扩大农产品流通。抓住国家取消高速公路鲜活农产品运输收费的有利契机，加快蔬菜生产基地建设，提高蔬菜品质，抓好储藏、流通等环节，扩大外销，蔬菜种植面积达到56万亩、增长33%，其中梅里斯区16万亩、增长60%。提高农民专业合作社的建设和管理水平，扩大农机、流通等专业合作社的合作领域和规模，新发展各类专业合作社800个。利用农机具购置补贴政策，新增千万元现代农机专业合作社50个。加强农业基础设施建设。加大资金投入和政策扶持力度，加快发展塑料大棚和日光温室等设施农业。推进“旱改水”，抓好西部地区节水灌溉和东部地区水资源开发，新增水田30万亩、节水喷灌50万亩。加快泰来抗旱引水工程、尼尔基下游配套灌区工程、音河和卫星两大灌区节水改造工程建设，争取花园水库工程早日开工建设。治理水土流失45万亩。扎实推进村镇建设。高标准搞好村镇布点规划、建设规划和产业发展规划。积极推进10个省级试点镇和14个市级试点镇规划建设，加快发展主导产业，完善公共基础设施，增强对人口的聚集能力，引导农村居民逐步向小城镇移居。坚持先行试点、循序渐进、尽力而为，切实抓好12个整乡（镇）推进和127个整村推进的示范乡（镇）村典型。建设农村公路500公里，植树造林1.39万公顷，发展秸秆固化、气化和沼气等清洁能源。加强就业技能培训，打造劳务品牌，搞好劳务对接，力争累计转移农村劳动力107万人，劳务收入增长10%以上。深入推进开发式扶贫，贫困人口下降8%以上。

（三）加快发展以现代服务业为重点的第三产业，提高支持一、二产业能力，增强区域中心城市服务功能。加快发展商贸物流业。借助交通基础设施全面升级的有利条件，建设连接黑河、满洲里两大口岸的区域物流枢纽基地。加快综合物流产业园区建设，年内完成园区管理中心、信息平台、货运枢纽工程、汽贸城等项目建设，促进传统物流业向现代物流业转变，带动绿色食品和小商品等产业发展。引进国内外知名的第三方物流企业，培育发展大型物流企业集团。推动各类专业市场提档升级。提高中心商业区建设水平，支持连锁经营、电子商务等新兴业态发展，促进便民商业网点向社区和农村延伸。提高餐饮娱乐业建设水平和服务档次。高质量办好绿博会（观鹤节）、小交会等重要会节，引进国内外客商来齐办展。扩大对外贸易，进出口总额增长15%以上。做大做强旅游业。高水准编制旅游业发展总体规划，提升旅游业发展水平。积极推进扎龙景区及齐扎公路旅游节点项目建设，年末前在景区内建成湿地博物馆、生态放飞广场、木栈道和水道延伸等项目；9月末前完成齐扎公路拓宽改造和温泉小镇“五通一平”，在沿线建设富裕老窖酒文化展示、飞鹤乳业生态观光牧场、北大仓酒文化产业园一期、绿色食品及旅游商品展销中心、飞碟射击基地等景点，打造精品旅游带。启动昂昂溪俄罗斯风情小镇建设，制定罗西亚大街文物保护和旅游开发规划，完成一期工程拆迁任务。完善碾子山区山水旅游和劳动湖、明月岛自然风光旅游项目，启动梅里斯区民族风情旅游开发项目。开设市区直通重点景区景点的观光巴士和旅游环线车。在劳动湖南扩项目中建设风味美食一条街。抓好7个市级重点旅游名镇规划建设，争取1-2个镇（乡）纳入省级旅游名镇。办好关东文化旅游节、冰雪游览会及各类特色旅游节庆活动。争取高档星级宾馆落地建设，提高旅游服务质量和接待能力。推动区域旅游联盟合作，打造黄金旅游线路，开发优质旅游产品，加强旅游宣传推介，旅游收入增长25%以上。大力支持金融业发展。积极引进金融机构设立地区总部或分支机构，力争引进浦发银行、哈尔滨银行。支持现有金融机构恢复和增设分支机构。搞好县级信用社向农村商业银行转型试点和金融机构设立村镇银行试点；力争小额贷款公司发展到20家、注册资本达到4亿元；规范整顿融资性担保公司，增强担保能力。做大做强城投公司，打造集融资、投资、资本运营和资源性开发为一体的城市经营平台。做好“十二五”及年度重大项目投融资规划。支持安泰生物在创业板上市，力争富尔农艺在美国纳斯达克主板上市，做好1-2家企业境外上市的培育工作。支持证券和保险业发展。加强征信系统建设，坚决打击高息集资、非法放贷、恶意逃债、非法和变相吸收公众存款等行为。积极发展文化产业。以旅游为线、文化为魂，用旅游带动文化产业繁荣，用文化促进旅游业振兴。发挥江桥抗战遗址公园、扎龙湿地、兴十四村、青年林场等影视基地作用，邀请知名影视制作公司和制片人拍摄反映齐齐哈尔文化特征、民族风情和地域风貌的影视作品。加快经营性文化事业单位转企改制，推进广播电视节目制播分离改革。整合文艺院团资源，拓展演艺市场。规划建设文化产业园，制定优惠政策吸引企业进驻，做大地方特色文化产业，做强动漫、策划等新兴文化创意产业。支持体育产业发展，鼓励企业和高校创办竞技体育团队，利用好现有体育场馆和设施，积极承办各类体育赛事。大力发展信息服务业，编制服务外包业发展规划，促进服务外包业加快发展。

（四）高水准搞好新区规划建设，全面加快旧城改造步伐，打造开阔疏朗、自然亲和、整洁优美的城市特色。深入落实“坚持以人为本、促进经济繁荣、铸造特色文化、突出滨水园林、改造新建并举、建设宜居鹤城”的城市建设思路，严格执行城市规划控制八项规定，认真实施城市建设各项规划，形成大建设、快改造、严管理的工作局面。高水准建设南苑新区。以国家高新区和南站建设为契机，抓好中央商务区、产业集中区、生活居住区、公共服务区等功能区规划建设，打造现代化、生态型、彰显文化内涵、体现独特风格的城市新区。把南苑新区作为今年全市城市建设的主战区，集中人力、物力、财力，加快推进南站、公路综合枢纽及广场建设，完成主要道路及配套设施，推进金融、教育、卫生等公共服务机构进驻。2012年完成90%的基础工程和公共服务工程建设，2013年基本建成功能完善的新城区，2015年形成承载40万人口的城市副中心。优化城市住房结构。以改善居民住房条件、满足各层面住房需求、提升城市整体形象为目标，搞好房屋开发和改造建设。今年市区开发建设商品房160万平方米，改造棚户区100万平方米、建设回迁楼140万平方米，建设廉租房1500套、6.75万平方米，建设公租房300套、1.8万平方米，配建一定比例的高档住宅，形成梯次供应的住房体系。加快“城中村”改造，建设实用美观的城市新居，改善“城中村”居民生活条件，统筹解决好农民就业、收入、保障等问题，实现农民向市民的转变。今年完成中心城区5个村改造任务。实施劳动湖南扩工程。劳动湖南扩是增强城市生态功能、改善城市环境的重大工程。今年6月末前完成全部拆迁安置任务，11月末前完成河道工程，同步搞好沿岸楼宇开发建设。到2012年绿博会前整体工程全面竣工，着力打造集生态、居住、休闲和旅游于一体的滨水景观带。搞好城市风貌改造。中华路是贯通中心城区东西的主要迎宾路，也是穿越卜奎古城区的道路。我们要用两年时间对中华路及两侧建筑实施路面、绿化升级和立面、亮化装扮的总体风貌改造，让三百年老城区的历史显露、文化呈现、绿荫绵延、交通顺畅。搞好广场、公园、滨水区域、主要道路两侧景观建设。提高牌匾广告规划设计水平，提升城市品位。完成沿江风光带建设规划，重点建设好浏园浴场和沿江景观节点。加大旧小区改造力度。对所有旧小区实施综合改造，年内改造完成1/3，力争3年全部改造完成，实现整洁、安全、美观、便民的目标。加快基础设施建设。完善路网建设，全面实现高速公路、齐富公路和跨江大桥建成通车。搞好城市综合交通规划，加密城区路网，完成卜奎大街南扩、中华东路、联通大道辅路等重点街路建设。搞好机场、火车站、汽车站、码头和站前广场、圣弥厄尔休闲文化广场等重点部位升级改造。提高原有热电联产供热能力，启动建设城南热电联产工程。抓好二次加压供水改造，提高供水能力和质量。加快南苑新区和各类产业园区供气设施建设。完成城区污水管网改造和垃圾处理场建设任务。做好嫩江西岸新区规划、水利设施筹建和生态保护工作。提高绿化水平。对5条街路、10个公园游园及广场绿地、10处街头转角进行绿化升级，提升城市公园文化品位，新增绿化面积18.4公顷，到2012年实现国家级园林城市目标。创新城市管理方式。上半年完成城市管理立法，实行城市管理相对集中行政处罚权。完善“两级政府、三级管理、四级网络”的城市管理体系，实现城市管理重心下移。借鉴黑河经验，确立环卫作业市场化运行机制，推行城市精细化管理，实现全方位覆盖和全时段监管。综合运用城市数字化信息系统，提高城市管理现代化水平。以市容市貌、违章建筑、城市交通和公共秩序综合治理为重点，大力开展专项整治，达到净化、亮化、绿化和美化目标，营造更加整洁有序的城市环境。巩固主要污染物减排成果。加大对松花江流域水污染防治“十一五”规划项目的后督察，确保污水处理设施切实发挥减排作用。强化各县（市）区政府、各企业的减排主体责任，对没有完成年度减排任务的实行严格问责。高质量完成全国污染源普查动态更新调查，为科学确定我市“十二五”主要污染物减排基数，全面完成减排任务奠定基础。

（五）大力发展各项社会事业，不断完善基本公共服务体系，进一步促进公共服务均等化。优先发展教育事业。认真实施全市教育发展规划，切实加大教育投入，促进教育均衡发展。建设标准化学校68所，改造危房校舍116.2万平方米，推进“校安工程”建设。积极支持民办教育发展，形成多元投入格局。加强师资队伍建设，创新用人机制，制定全员培训规划，提高教师素质和教育教学质量。深入实施课程改革，加快教育信息化进程，减轻学生课业负担，推进素质教育。大力发展学前教育，制定学前教育三年发展规划，年内改扩建和新建乡镇、村级幼儿园230所和县（市）区级公办实验幼儿园15所。推行集中办学和寄宿制办学模式，促进农村义务教育发展。加快发展高中阶段教育，普及率达到85.5%，市直属重点高中自费生比例控制在30%以下。提高职业教育办学质量，根据市场需求搞好专业设置，为学生就业和服务社会打好基础。统筹用好就业培训资金，搞好职业教育公共实训基地建设。支持高等教育发展，加强校地、校企合作。切实提高医疗卫生服务水平。以打造区域医疗救治服务中心为目标，加强三级医院内涵建设，突出专科医院特色，促进综合医院更强、专科医院更优。抓好第一医院、中医院、结核医院扩能提质和县区医院建设。加快医疗人才队伍建设，提高医疗救治能力和服务水平，增强对周边地区的吸引力，力争市外患者就诊率提高8个百分点。完成医药卫生体制改革三年任务，解决群众看病难、看病贵问题。推进社区卫生与城镇职工、居民医疗保险和医疗救助相结合，构筑以社区服务为基础的新型城市医疗卫生服务体系。加强农村医疗人才和卫生院所建设，完善新农合服务体系，构建信息化平台，实现“一卡通”和远程会诊。扩大公共卫生服务和计划免疫规划范围，搞好重大疫病防治和突发公共卫生事件处置。着力提高公共文化服务能力。坚持保护与开发并重，深入挖掘历史文化资源，为评定国家级历史文化名城创造条件。做好昂昂溪古文化遗址发掘保护工作，加快史前文化遗址博物馆建设。推进黑龙江将军府复建工作，对重要的历史文化建筑进行修缮保护。整顿规范文化市场秩序，严厉打击违法音像制品和非法出版物经营活动。加强城乡文化、体育基础设施建设，深入开展群众性文体活动。进一步做好人口和计划生育工作。

（六）全面推进民生工程，认真办好惠民实事，努力增进人民福祉。促进和扩大社会就业。实施更加积极的就业政策，抓好省级创业型城市建设，落实好社会保险、职业介绍、技能培训等补贴政策，深入推进创业带就业工作。健全城乡公共就业服务体系，完善就业信息发布、资源共享机制，发挥好人力资源市场作用，建立失业动态监测机制，深入推进援企稳岗。加强职业技能培训，增强创业就业能力。抓好高校毕业生就业，开发公益性岗位4600个，加大对零就业家庭援助力度，帮助4.9万名就业困难人员实现就业。实现城镇新增就业9万人，下岗失业人员再就业7.5万人。努力提高社会保障水平。扩大养老保险覆盖面，抓好第二批新型农村社会养老保险试点，解决好农民养老和城镇无业老人养老保障问题。抓好城镇化进程中养老保障省级试点，切实保障失地农民利益。完善城镇居民基本医疗保险市级统筹，加强医疗保险城乡一体化管理，着力解决关闭破产集体企业和困难企业职工医疗保险问题。继续推进城镇居民门诊统筹，深入实施参保人员转诊转院试行办法，适时开展城镇职工门诊统筹，进一步方便群众就医。深入推进新农合，提高农民医疗保障标准。做好失业、工伤、生育保险工作。切实加强对贫困群众的救助，着力解决低保及低保边缘人群的生活问题；加强“五保”规范化管理，提高集中供养率；发放廉租住房补贴3084万元；加大对贫困革命老区扶持力度。全力维护社会和谐稳定。抓好社会治安防控基础建设，加强中心城区公安民警、武警联勤和公益岗位人员治安巡逻，加大楼宇门安装维修力度，推进3G无线视频监控系统建设，提升信息化管控水平，构建群防群治体系。加强公安指挥中心建设，完善应急处置机制，提高应急服务水平和处置突发事件能力。加大隐患排查力度，切实加强生产、消防和道路交通安全工作。开展查处取缔无证无照经营专项整治行动，加大对假冒伪劣产品打击力度，加强乳制品等食品药品生产、销售环节的监管，确保质量安全。强化供热温度、供气安全和供水质量监管，确保达到规定标准，对供热不达标的企业进行整顿清理。落实信访责任制，坚持领导干部信访接待日制度，搞好联合接访大厅建设，整合投诉电话和网络平台，加强市长公开电话受理工作，及时有效地解决各类信访问题。做好“六五普法”和人民调解、法律援助、安置帮教等工作，加快依法治市进程。有效稳控物价，保障群众基本生活。提高社区建设水平。加强基层政权建设，提高社区工作人员素质和待遇，推进居（村）务公开。认真实施社区公益用房三年发展规划，加大清缴开发商拖欠公益用房力度，完成70%的建设任务。深入开展和谐社区建设示范单位创建活动，加快发展社区居家养老等特色服务项目，建立更多的以兴趣为中心的社团组织，提高社区居民快乐指数。继续做好“国际老年友好城市”巩固提高工作，打造“孝亲敬老之城”。

根据社会调查和群众意愿，今年办好20件实事。一是改善公共交通状况，将客流量大的公交线路冬季延时至20点，夏季延时至21点，合理调整上下班高峰运营频率。二是搞好停车场（位）建设，新建小区严格按照城市规划控制规定的要求建设停车场，未建停车设施及停车设施明显不足的已建成小区，有条件的也要新建扩建地下停车设施，公共建筑要按照使用功能配建停车场。三是加大城区路网密度，打通“断头路”，拓宽“卡脖路”，改造次干道和巷道，在规模较大的小区增加车辆进出口，并对超大的小区分区处理，形成道路网格化，保障交通顺畅。四是搞好棚户区改造，力争1.2万户居民迁入新居。五是改造农村泥草房3.6万户。六是改造低温楼40万平方米。七是抓好廉租房、公租房建设和廉租住房补贴发放工作。八是加强小区物业和环境管理，实现物业收费、服务内容和服务标准三公开，在中心城区有物业覆盖的小区全部建立业主委员会，并抓好小区亮化、绿化、硬化和美化工作。九是解决行人如厕难问题，中心城区新增环保公厕10座、大型移动式环保公厕2座。十是确保中小学生用餐质量和在校安全，通过招标方式选择餐饮企业为学生配送午餐，依法取缔校园周边流动摊点；加强中小学安保力量和送子车管理，规范校园周边治安和交通秩序，实行错时放学，减轻交通压力。十一是加强食品安全监管，严厉查处加工、倒卖、使用地沟油和不符合质量标准的食品添加剂等违法行为，运用信息化手段加大对饮食行业餐具消毒的监管力度。十二是抓好农村饮水工程建设，解决10.5万人饮水安全问题。十三是实施创业带就业工程，为7000人发放小额担保贷款2亿元。十四是提高被征地农民养老保险金待遇标准，力争纳入城镇社保体系管理，进入省级统筹。十五是扩大医疗保险覆盖面，解决14.8万名关闭破产集体企业和其他困难企业退休人员医保问题。十六是实现基本药物零差价销售全覆盖，社区卫生服务机构覆盖人口增加4万人，覆盖率达到100%。十七是做好贫困残疾人医疗康复和住房救助工作。十八是在劳动湖沿岸建设全民健身长廊。十九是加快民营托老所建设，中心城区新建100张以上床位的托老所5处，提升服务质量。二十是加快社区文化娱乐场所建设，城区1/3以上的社区公益用房使用面积要达到300平方米，并按70%以上的比例设置居民文化娱乐空间。

各位代表，做好新形势下的政府工作，必须切实加强政府自身建设。着力提高政府全体公务人员的思想、政治和业务素质，增强工作的主动性和创造性，焕发干事创业的激情，求真务实不虚假，坚韧不拔不气馁，勤勉敬业不懒惰，认真负责不推拖，持之以恒不折腾。着力推进决策的科学化、民主化，自觉接受人大的法律监督、工作监督和政协的民主监督，广泛听取社会各界的意见和建议，着眼长远发展，关注社会热点，强化市场意识，讲求投资效益，维护公平正义，确保依法行政。着力提高执行力和落实力，科学谋划发展，善抓主要矛盾，把握关键环节，突破重点难点，明确目标责任，强化督办检查，用真招实法解决发展中的实际问题。着力强化服务职能，扩大政务公开范围，办好政府门户网站，推行网上办事、网上互动，简化审批服务流程，对重点项目实行监察、审计部门全程跟踪督导，扎实搞好行风评议，用最便捷的服务为项目建设赢得宝贵时间。着力加强廉政建设，坚持用制度管钱、管人、管事，从源头上预防腐败现象滋生。

各位代表！我们已经踏上“十二五”的新征程。保持好强劲的发展势头，奋力推进经济社会的新跨越，建设一个富庶、宜居、和谐的新鹤城，是群众的期盼，也是时代的要求。让我们在市委的领导下，万众一心，埋头苦干，为创造全市人民更加美好幸福的生活而努力奋斗！
